# Integration and segregation of large-scale brain networks during short-term task automatization

**DOI:** 10.1038/ncomms13217

**Published:** 2016-11-03

**Authors:** Holger Mohr, Uta Wolfensteller, Richard F. Betzel, Bratislav Mišić, Olaf Sporns, Jonas Richiardi, Hannes Ruge

**Affiliations:** 1 Department of Psychology, Technische Universität Dresden, Dresden 01069, Germany; 2 Department of Psychological and Brain Sciences, Indiana University, Bloomington, Indiana 47405, USA; 3 Department of Bioengineering, University of Pennsylvania, Philadelphia, Pennsylvania 19104, USA; 4 McConnell Brain Imaging Centre, Montreal Neurological Institute, McGill University, Montreal, H3A 2B4 Quebec, Canada; 5 Indiana University Network Science Institute, Indiana University, Bloomington, Indiana 47405, USA; 6 Department of Neuroscience, Laboratory of Neurology and Imaging of Cognition, University of Geneva, Geneva 1202, Switzerland

## Abstract

The human brain is organized into large-scale functional networks that can flexibly reconfigure their connectivity patterns, supporting both rapid adaptive control and long-term learning processes. However, it has remained unclear how short-term network dynamics support the rapid transformation of instructions into fluent behaviour. Comparing fMRI data of a learning sample (*N*=70) with a control sample (*N*=67), we find that increasingly efficient task processing during short-term practice is associated with a reorganization of large-scale network interactions. Practice-related efficiency gains are facilitated by enhanced coupling between the cingulo-opercular network and the dorsal attention network. Simultaneously, short-term task automatization is accompanied by decreasing activation of the fronto-parietal network, indicating a release of high-level cognitive control, and a segregation of the default mode network from task-related networks. These findings suggest that short-term task automatization is enabled by the brain's ability to rapidly reconfigure its large-scale network organization involving complementary integration and segregation processes.

## Abstract (web summary)

Humans can quickly learn to efficiently execute tasks yet how the brain activity is dynamically reconfigured during this process remains unknown. Here the authors demonstrate that large-scale functional brain networks are reorganized flexibly to support rapid task automation.

The ability to adapt behaviour based on past experiences and future goals is an important characteristic of humans and other animals. These adaptive processes can be observed across different timescales, ranging from seconds to years. Recently, interest has grown in understanding how especially humans can flexibly and rapidly transform instructed rules into goal-directed behaviour^1^,^2^,^3^. This ability to instantaneously transform abstract information into adequate behaviour with high fidelity stands in contrast to long-term learning, where behavioural patterns are repeatedly practiced over hundreds or thousands of trials to achieve mastery^4^.

To understand how those adaptive processes are implemented in the human brain, a novel approach has emerged over the past years, in which neuroimaging data of the human brain are modelled as a set of large-scale networks^5^,^6^. The general hypothesis of this approach is that neural responses to a momentary challenge (for example, stimulus or task) are not only reflected by a change of neural activity in certain regions of the brain but also by a global reorganization of connectivity patterns^4^,^7^,^8^,^9^.

Applying this approach to rapidly changing task rules, Cole *et al*.^2^ showed that flexible adaptation to these task rules is associated with variable connectivity patterns between a specific cognitive control network, the fronto-parietal network (FPN) and other large-scale networks. Cole *et al*. also compared novel task rules with practiced task rules, where practice was performed in 2 h sessions before scanning, and found stable compositional representations of task rules in FPN connectivity patterns^2^.

In contrast, Bassett *et al*. investigated the effects of long-term training of motor sequences on the organization of large-scale brain networks^4^,^7^,^10^. Bassett *et al*. demonstrated that long-term learning is associated with enhanced segregation of visual and motor areas from other cognitive control networks, reflecting increased autonomy of basal sensory and motor networks.

Short-term transformation of instructed rules into fluent task processing as examined in the present study is distinctly different from the non-repetitive, flexible implementation of novel task rules on the one hand and long-term learning on the other hand. We all have experienced that a limited amount of novel task practice can quickly lead to a subjectively easier and more efficient task performance. Until now, it has remained unclear how those efficiency gains are achieved by the human brain. By investigating systematic dynamic changes of large-scale connectivity patterns, we aimed to understand how short-term task automatization is implemented in the brain.

More specifically, we were interested in finding out whether short-term practice of instructed stimulus–response (S–R) rules would have a measurable effect on large-scale connectivity patterns, and if so, whether it would be associated with changes between high-level cognitive control networks or more low-level visual and motor networks. Adaptation to a certain repetitive task might be associated with stronger integration and segregation of brain networks^11^ in the sense that networks necessary to process relevant information become increasingly coupled, as shown before^8^, and networks that do not contribute to task performance are segregated from task-relevant networks^12^.

Motivated by the aforementioned results, we analysed the functional magnetic resonance imaging (fMRI) data of the instruction-based visuo-motor learning task^13^ to see how large-scale brain networks dynamically change their connectivity patterns during short-term practice of S–R rules. In this task (see also Fig. 1), subjects were presented with an instruction screen for 10 s, where they had to memorize S–R associations for four visual symbols and two motor responses. After the instruction period, a sequence of single symbols was presented, and subjects had to respond to them as instructed before. Each of the symbols appeared eight times (counting only correct trials), leading to a practice phase duration of ∼90 s. Instruction and practice were repeated 20 times using novel symbols each time to increase statistical power. Subjects performed the task inside an MRI scanner (*N*=70). We were then interested in seeing how the large-scale brain connectivity pattern during the first third of the practice phase (in the following termed early practice, ∼30 s) differed from the connectivity pattern of the last third of the practice phase (in the following termed late practice, ∼30 s, see also Methods for more details).

Importantly, short-term practice of instructed S–R rules is conceptually different from reinforcement learning of S–R associations^14^,^15^. In a trial-and-error learning set-up, subjects start to perform the learning task without prior knowledge about S–R contingencies, and learning is traditionally measured in terms of decreasing error rates, typically starting at chance level^16^,^17^,^18^. In contrast, instruction of S–R associations before response execution should induce low initial error rates. Behaviourally, one would then expect to find a speed-up of response times (RTs) in correct trials in combination with stable or further decreasing error rates, indicating an increasingly efficient transformation of stimulus input into motor output across practice. In addition to behavioural differences, it has been shown that instruction-based learning and trial-and-error learning induce dissociable neural activation and connectivity patterns^19^,^20^.

Using this kind of experimental contrast, that is, comparing the first part of a practice block with the last part of a practice block, one needs to carefully control for practice-unrelated effects, since it has been shown before that general, task-independent dynamic effects can occur during a task block^21^. In addition, since novel symbols were used for each of the 20 learning sets, parts of the adaptation process might reflect increasingly efficient visual processing of the specific symbols. To control for adaptive processes unrelated to practicing S–R rules, we acquired a control sample (*N*=67), where stimulus material and temporal structure were identical to the learning task, but practicing S–R associations was prevented. This was achieved by replacing the task requirements during the practice phase. Instead of practicing instructed S–R rules, subjects of the control sample were required to perform a 1-back working memory task with randomized left/right manual responses to indicate a 1-back match or mismatch. The rationale behind the control task is discussed in more detail in the Methods.

For network construction, we used a set of network nodes that have been demonstrated to be representative for the networks under investigation^22^,^23^. Of these originally 264 nodes, 227 nodes have been assigned to 10 well-established large-scale networks, comprising low-level input and output networks (visual, auditory and sensorimotor networks), subcortical nodes, the default mode network (DMN), ventral and dorsal attention networks (VAN and DAN), and cognitive control networks (FPN, cingulo-opercular network (CON), salience network (SAN)), using a network community detection algorithm^2^,^23^. After quality control, 222 nodes were included into connectivity analyses, and connectivity values for all edges between the 222 nodes were computed and averaged to obtain mean connectivity values between and within networks for early and late practice, respectively. The change of activation and connectivity from early to late practice was compared between the learning sample and the control sample to identify effects specific to S–R rule practice.

Based on these large-scale activation and connectivity analyses, we demonstrate that short-term practice of instructed S–R rules is associated with a reorganization of large-scale network interactions. Specifically, we find that task automatization is facilitated by enhanced coupling between the CON and DAN, and complemented by decreasing activation within the FPN. In contrast, the DMN shows increasing activation and segregation from task-related networks, indicating a shift of resources towards task-unrelated processes. These findings support an integrative view of the brain, suggesting that task-dependent plasticity is not sufficiently reflected by locally isolated activation changes, but is instead characterized by global activation and connectivity changes between and within several large functional networks.

## Results

### Behavioural results

Behaviourally, we used relative changes of RTs of correctly performed trials as an indicator for a practice-related efficiency increase (see also Methods). As expected, a comparison of relative RT changes between the learning and control sample revealed that practicing S–R rules led to a significantly larger relative speed-up of RTs than performing the 1-back task (*P*=0.00004, *t*=4.3, df=135, two-sample *t*-test, see also Supplementary Fig. 1), with on average 6.4% RT decrease for the learning task and 3.5% decrease for the control task. Error rate changes also differed significantly between the learning sample and the control sample, for details see Supplementary Fig. 1.

### Activation changes

**Figure 2:**
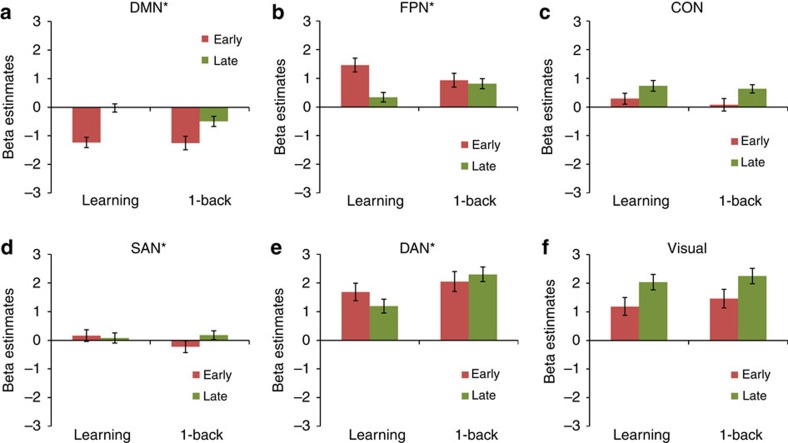
Mean activations for 6 of the 10 networks for early and late practice and for the learning and control sample respectively. (**a**–**f**) Asterisk indicates a significant difference between the learning sample (*N*=70) and the control sample (*N*=67) in terms of activation changes from early to late practice, that is, a two-sided two-sample *t*-test (learning (late–early) versus control (late–early)) was significant (*P*<0.05) after Bonferroni correction for 10 tests. Black lines represent 95% confidence intervals. Activations of the four remaining networks can be found in Supplementary Fig. 2. *P* values and *t*-values for all 10 tests can be found in Supplementary Table 1. CON, cingulo-opercular network; DAN, dorsal attention network; DMN, default mode network; FPN, fronto-parietal network; SAN, salience network.

In the FPN, activation decreased during the practice of S–R rules but not during the 1-back task (Bonferroni-corrected *P*=5 × 10^−9^, *t*=−6.7, df=135, two-sample *t*-test, corrected for 10 tests), see also Fig. 2. Moreover, DAN activation decreased from early to late practice in the learning sample, but not in the control sample (Bonferroni-corrected *P*=6 × 10^−5^, *t*=−4.7, df=135). In contrast, for the DMN increasing activation was observed across practice in both samples, with a larger increase in the learning sample than in the control sample (Bonferroni-corrected *P*=0.013, *t*=3.3, df=135). Activation in the CON increased in both samples without a significant difference between the samples (uncorrected *P*=0.3, *t*=−1.0, df=135). Furthermore, while a significant difference between the two samples was found for the SAN (Bonferroni-corrected *P*=0.0008, *t*=−4.1, df=135), testing early against late practice in the learning sample alone did not confirm a practice-related effect (uncorrected *P*=0.3, *t*=−1.0, df=69, one-sample *t*-test). Activation in the visual network increased in both samples without a significant difference between the samples (uncorrected *P*=0.6, *t*=0.5, df=135). The four remaining networks (auditory and subcortical networks, sensorimotor network, and VAN) did not show significant differences between the samples; respective bar plots can be found in Supplementary Fig. 2. In Supplementary Table 1, test results are reported for all 10 networks, comprising two-sample and one-sample tests.

### Connectivity changes

**Figure 3:**
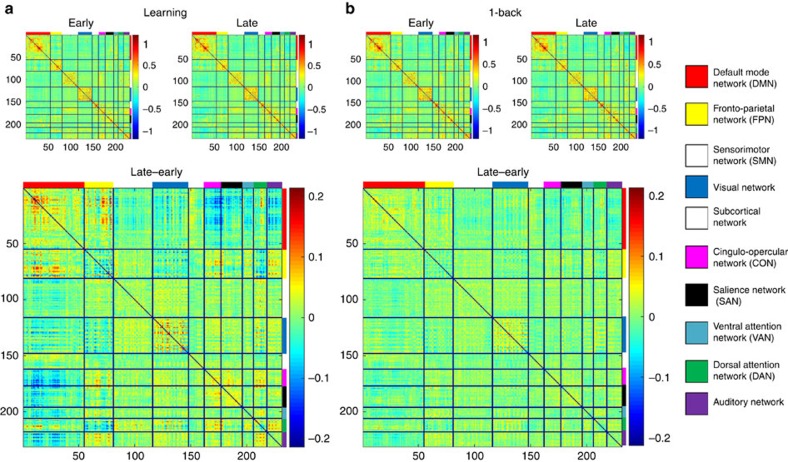
Mean connectivity matrices and practice-related changes in connectivity for the learning sample (*N*=70) and the control sample (*N*=67). (**a**) Mean connectivity matrices for the learning sample. In the top row, mean values of Fisher *z*-transformed correlation coefficients between the 222 nodes are shown for early and late practice separately. Colour scales range from −1.1 to +1.1. Nodes belonging to the same network were grouped together. In the bottom row, connectivity changes from early to late practice are depicted. Note that colours were rescaled to represent values between −0.2 and +0.2. (**b**) Same matrices for the control sample. For a comparison of between-network and within-network connections, see Supplementary Fig. 3.

Connectivity matrices for the 222 nodes, sorted by networks, reproduced the typical community structure of high connectivity within networks (squares on the diagonal in the top row of Fig. 3) and low connectivity between networks on average (off-diagonal rectangles in the top row of Fig. 3) for early and late practice in both samples (see also Supplementary Fig. 3).

**Figure 4:**
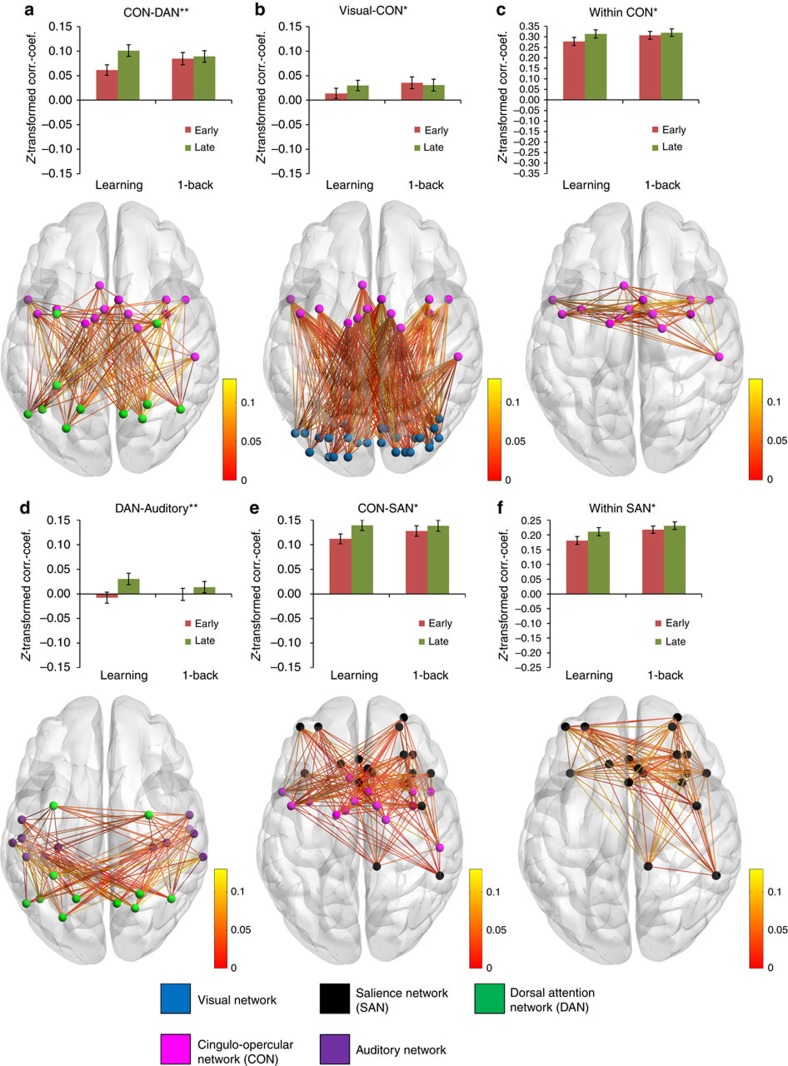
Increasing connectivity between or within networks from early to late practice. Bar plots show mean connectivity values between or within networks for early and late practice in both samples. Asterisks indicate that increases across practice in the learning sample (*N*=70) were significantly larger than in the control sample (*N*=67), with significance levels (**) for Bonferroni correction *P*<0.05 and (*) indicating FDR correction *P*<0.05, using two-sided two-sample *t*-tests. Black lines represent 95% confidence intervals. *P* values and *t*-values for all 55 between/within-network tests can be found in Supplementary Table 2. Brain images show edge-wise connectivity changes from early to late practice in the learning sample. Edge colours represent *z*-transformed correlation coefficient values. For clarity, edges with negative values are not shown. For 3D visualization, see also online Supplementary Video Material, videos 1–6. (**a**,**b**,**d**,**e**) Mean connectivity changes between networks. (**c**,**f**) Mean connectivity changes within networks.

**Figure 5:**
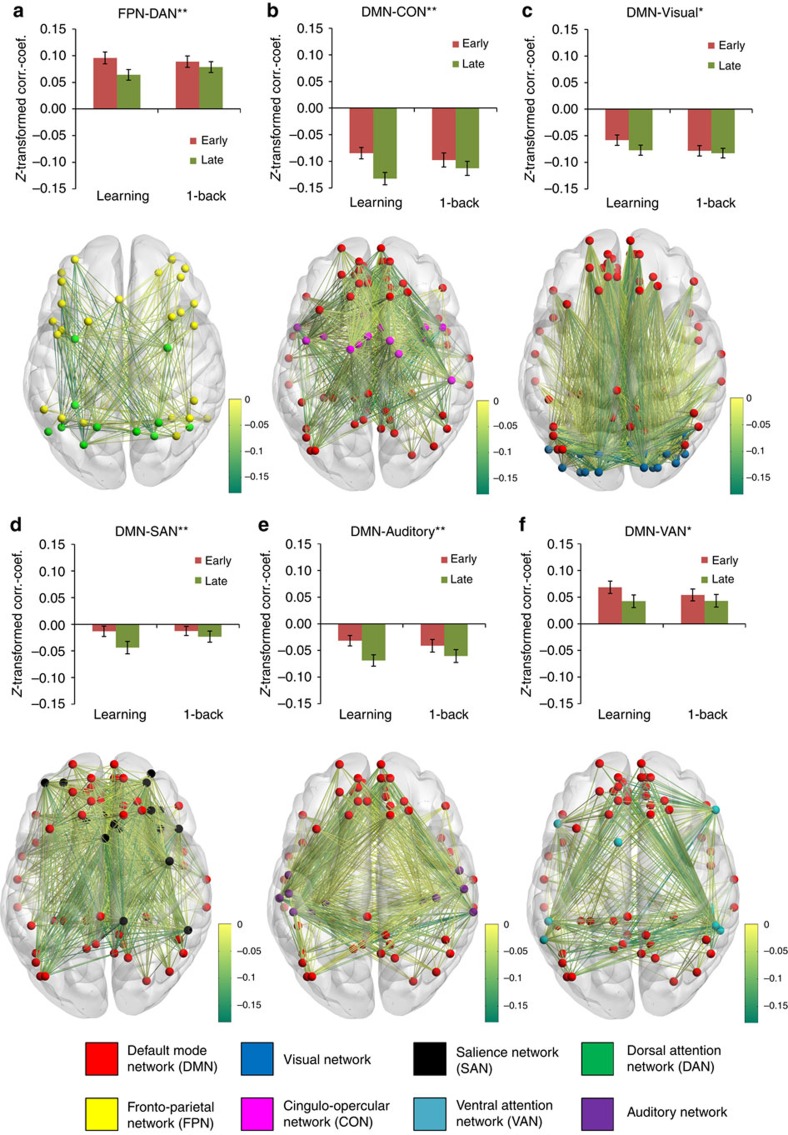
Decreasing connectivity between networks from early to late practice. Bar plots show mean connectivity values between networks for early and late practice in both samples. Asterisks indicate that decreases across practice in the learning sample (*N*=70) were significantly larger than in the control sample (*N*=67), with significance levels (**) for Bonferroni correction *P*<0.05 and (*) indicating FDR correction *P*<0.05, using two-sided two-sample *t*-tests. Black lines represent 95% confidence intervals. *P* values and *t*-values for all 55 between/within-network tests can be found in Supplementary Table 2. Brain images show edge-wise connectivity changes from early to late practice in the learning sample. Edge colours represent *z*-transformed correlation coefficient values. For clarity, edges with positive values are not shown. For 3D visualization, see also online Supplementary Video Material, videos 7–12. (**a**) Mean connectivity change between the FPN and DAN. (**b**–**f**) Mean connectivity changes between the DMN and task-related networks.

Comparing connectivity changes from early to late practice between the two samples revealed that practicing S–R rules was associated with a larger increase in connectivity between the CON and DAN (Bonferroni-corrected *P*=2 × 10^−6^, *t*=5.8, df=135, two-sample *t*-test, correction for 55 tests), and between the DAN and auditory network (Bonferroni-corrected *P*=0.015, *t*=3.7, df=135). Respective bar plots and three-dimensional (3D) visualizations of brain images are depicted in Fig. 4. A larger decrease in connectivity in the learning sample than in the control sample was observed between the DMN and CON (Bonferroni-corrected *P*=2 × 10^−6^, *t*=−5.8, df=135), between the DMN and SAN (Bonferroni-corrected *P*=0.009, *t*=−3.9, df=135), between the DMN and auditory network (Bonferroni-corrected *P*=0.015, *t*=−3.7, df=135), and between the FPN and DAN (Bonferroni-corrected *P*=0.025, *t*=−3.6, df=135). See Fig. 5 for respective bar plots and 3D visualizations of brain images.

Using the more sensitive false-discovery rate (FDR) correction for multiple testing further revealed that practicing S–R rules was associated with increased coupling between the CON and visual network (FDR-corrected *P*=0.026, *t*=3.0, df=135), and between the CON and SAN (FDR-corrected *P*=0.023, *t*=3.0, df=135). In addition, increasing connectivity within networks was found for the CON (FDR-corrected *P*=0.025, *t*=2.9, df=135) and SAN (FDR-corrected *P*=0.028, *t*=2.8, df=135). Moreover, larger segregation of the DMN from the visual network (FDR-corrected *P*=0.045, *t*=−2.7, df=135) and VAN (FDR-corrected *P*=0.041, *t*=−2.6, df=135) was observed for the learning sample than for the control sample.

Testing early against late practice in the learning sample alone confirmed that the reported effects were indeed practice-related. See also Supplementary Table 2 for the results on all 55 tests, both between and within samples. Videos of the 3D visualizations of the brain images are available online (Supplementary Video Material).

As further elaborated in the Discussion, the segregation of the DMN from other networks combined with an activation increase points towards a dissociation of task-relevant processes and task-unrelated processes, which seem to run increasingly in parallel. While this finding provides interesting insights regarding the dynamics of the DMN during practice, it might be rather a by-product of increased efficiency than its original source. Instead, it seems more plausible that the efficiency increase originates from enhanced coupling between task-related networks. Hence, in the following, we focused our further analyses on the connections between the CON and DAN, which displayed the largest practice-related increase in connectivity.

### Subdivision of CON and DAN

Both the CON and DAN were composed of network nodes from several remote regions of the brain. To investigate whether the average practice-related increase in functional connectivity between the CON and DAN was driven by a specific subcluster of network nodes, or, alternatively, reflected a broadly distributed increase, we divided the CON and DAN into spatially defined subnetworks. More precisely, we divided the CON into four subclusters consisting of anterior insula/frontal operculum, supplementary motor area (SMA)/dorsal anterior cingulate cortex, midcingulate cortex and supramarginal gyrus, and the DAN into four subclusters consisting of precuneus/parietal cortex, temporal lobe, frontal eye fields and occipital cortex. We then averaged connectivity values, that is, the change of connectivity from early to late practice, over all edges connecting pairs of subclusters, and subtracted respective values of the control sample from the values of the learning sample to distill the practice-related increase. The obtained distribution of connectivity change values was found to be normal (*P*=0.58, Shapiro–Wilk test), see Supplementary Fig. 4. This finding shows that the larger connectivity change for practice compared with control between CON and DAN was not driven by increasing connectivity between specific sets of regions, but instead the mean increase reflects a broad increase across regional subclusters.

### Connectivity and RTs

**Figure 6:**
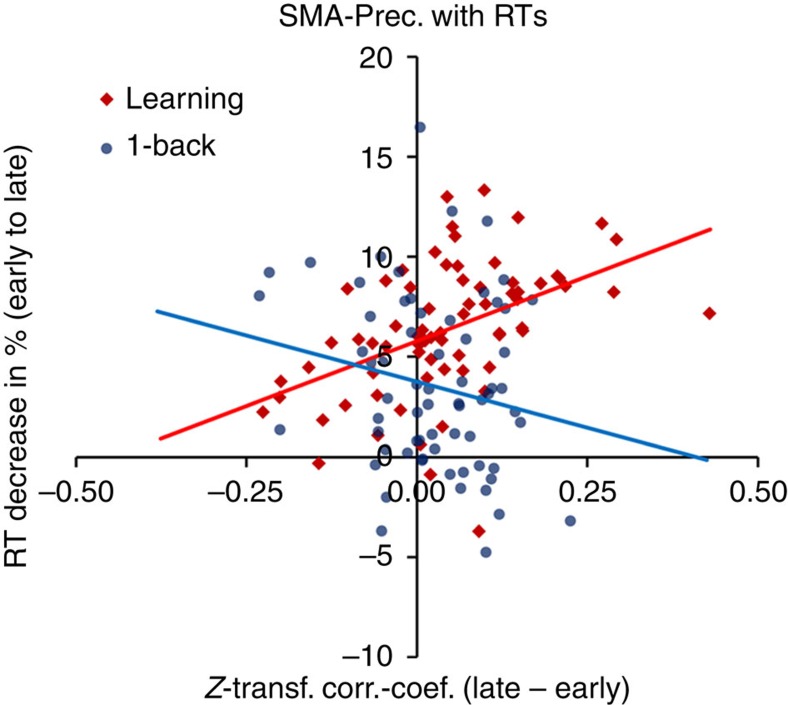
Correlation of connectivity change with response time (RT) decrease. The plot shows connectivity change between a node of the CON (within the SMA) and a node of the DAN (within the precuneus) on the x-axis and percentage of RT decrease on the y-axis. For the learning task, a higher increase in connectivity predicted a larger decrease in RTs during practice (Pearson *r*=0.47, *z*=4.2, Bonferroni-corrected *P*=0.004). The difference between the learning sample (*N*=70) and the control sample (*N*=67) was also significant (z=4.1, Bonferroni-corrected *P*=0.008). No significant correlation was found for the control sample (*r*=−0.20, *z*=−1.6, uncorrected *P*=0.11). Data points represent values of individual subjects. For activation and connectivity results for the two nodes, see Supplementary Fig. 5. RT, response time; SMA, supplementary motor area; Prec., precuneus; CON, cingulo-opercular network; DAN, dorsal attention network.

Further investigating the CON–DAN connections, we examined whether interindividual differences in connectivity change between the CON and DAN predicted interindividual differences in RT change in the learning sample. This was not the case for mean connectivity values between the two networks (*P*=0.24, *z*=−1.2, two-sided test for different Pearson's *r* in the learning sample versus the control sample). We hypothesized that interindividual differences might have been lost by averaging connectivity change across edges. Hence, we tested the connectivity change of all pairwise connections between the CON and DAN separately on their correlation with RTs. Indeed, the connection between a precuneus-based (Montreal Neurological Institute (MNI)=10, −62, 61) and an SMA-based (MNI=−16, −5, 71) node was found to be highly predictive for RT speed-up in the learning task (Bonferroni-corrected *P*=0.008, *z*=4.1, two-sided test for different Pearson's *r* in the learning task versus the control task, corrected for 154 tests), depicted in Fig. 6. Importantly, to confirm that this difference in correlation between the two samples reflected a practice-related RT speed-up specific to the learning sample, we tested the correlation in the learning sample against zero, which was again significant (*r*=0.47, *z*=4.2, Bonferroni-corrected *P*=0.004). In contrast, no significant correlation was found for the control sample (*r*=−0.20, *z*=−1.6, uncorrected *P*=0.11). For activation and connectivity results for the two nodes, see Supplementary Fig. 5.

## Discussion

Comparing large-scale connectivity and activation dynamics between a learning sample and a control sample, we found that short-term practice of S–R rules led to decreasing activation within the FPN and DAN, accompanied by enhanced coupling among several task-related networks, most prominently between the CON and DAN. Practicing S–R rules was further associated with increasing activation within the DMN combined with a segregation of the DMN from several task-related networks. Moreover, connectivity increase between two nodes within the SMA (CON) and precuneus (DAN) correlated with practice-related efficiency increase.

Generally, the idea that practice could be associated with increased connectivity between task-processing regions has been discussed before^24^,^25^. Recently, Bassett *et al*. investigated long-term motor sequence learning in the context of large-scale networks and found increased autonomy of visual and sensorimotor networks, accompanied by a practice-related release of large portions of the frontal and temporal lobe^4^. In the rapid learning paradigm presented here, practice was limited to eight repetition levels. The associated short-term learning processes were characterized by distinct connectivity changes among high-level cognitive control networks as well as primary sensory networks. In the next sections, we will discuss our findings, specifically the FPN activation decrease and decoupling from the DAN, the connectivity increase between CON and DAN, possible interactions with the SAN, and the role of the DMN.

Activation decrease observed during practice is typically interpreted as increasingly efficient task processing in the respective region, possibly combined with a reorganization of processing pathways involving a shift of processing streams to other regions^24^. The drop of activation within the FPN in the learning sample, together with the reduced functional connectivity between the FPN and DAN, indicate that high-level computations of correct responses were only necessary at the beginning of the practice phase. It has been shown before that immediately after the instruction of S–R rules, the resulting representations of these rules are being held in working memory^26^. Hence, at the beginning of practice, abstract representations about the correct response had to be retrieved from working memory and transformed into an actual motor response involving high-level processing^13^,^27^. Working memory-related processes typically recruit the FPN^28^,^29^, which was also observed in the control sample of the current study. In addition, direct evidence for an implication of the FPN in rule processing has been presented before, indicating that difficult S–R rules increase FPN activation, compared with easier rules^30^. Consistent with our results, an activation decrease in parts of the FPN combined with an activation increase in parts of the DMN has also been observed for longer practice sessions^31^. In contrast, in the control task, online response computation had to be repeatedly performed in each trial, since the comparison of the current and preceding symbol and the mapping of equal/unequal to left/right-hand responses changed from trial to trial. Accordingly, FPN activation remained stable during the 1-back task. Additional to the activation decrease, the segregation of the FPN from the DAN provides further evidence that the FPN disengaged from S–R transformation during practice. Specifically, as high-level control was provided by the FPN primarily during the early practice phase, the connectivity decrease indicates that the DAN became less dependent on abstract, high-level S–R representations. Instead, stimulus inputs seemed to be transformed into associated motor outputs more directly, as the DAN did not only reduce functional connectivity with the FPN, but also increased connectivity with the CON, which we will discuss in the following section.

As practice proceeded, retrieval of abstract S–R representations and general guidance by the FPN was needed to a lesser extent. Instead, visual inputs were transformed into appropriate motor responses more directly, supported by increasing functional coupling between the CON and DAN. The notion that this short-term task automatization and the associated efficiency increase were indeed facilitated by a strengthened coupling between the CON and DAN was supported by the predictive power of connectivity strength between two specific network nodes within SMA and precuneus for RT acceleration. In addition, functional connectivity increased between the CON and the visual network, indicating that low-level stimulus representations were integrated more directly into the response selection process during late practice.

While recent studies have begun to characterize task-dependent modulations of large-scale functional networks^8^,^9^,^32^,^33^,^34^,^35^,^36^, little is known about the specific functional relevance of task-dependent connectivity changes between the CON and DAN. The DAN itself has been associated with short-term memory of visual features and also with linking relevant visual stimulus features to responses^33^,^37^,^38^, that is, functions that are also implied in the instruction-based learning task. In a carefully controlled design, Rushworth *et al*.^38^ showed that rule switching activated the posterior parietal cortex, indicating that S–R transformation could be processed in this region. In addition, posterior parietal cortex connectivity has been associated with faster learning rates^39^. Together, these findings suggest that the activation decrease in the DAN reflects an increasingly efficient S–R transformation in this network.

The CON is mainly known for its tonic activation during task blocks, and supposed to maintain a stable, across-trial representation of the task set and to support downstream sensorimotor processes^21^,^40^. Intriguingly, Dosenbach *et al*.^41^ proposed that ‘feedback signals received by the CON, rather than causing immediate adjustments, might perhaps be integrated over many repetitions in a more protracted iterative fashion'. Corroborating this hypothesis, practice of S–R transformations led to a more distinct connectivity profile of the CON, involving strengthened pathways to the DAN, SAN and visual network, as well as higher within-network connectivity and better segregation from the DMN. Concerning the link between precuneus and SMA, which predicted practice-related RT decrease, a recent MEG study provided converging evidence for an essential involvement of this connection in S–R execution^42^. In this study, it was shown that Brodmann area 7 (precuneus/parietal cortex) exerts influence on Brodmann area 6 (SMA/premotor cortex) during the performance of S–R mappings, supporting the conclusion that stimulus inputs are first processed within the DAN and then transformed into appropriate motor responses via the DAN–CON pathway.

Besides displaying increasing connectivity with the CON, the DAN also strengthened its coupling with the auditory network. As no auditory stimuli were presented during the task, this connectivity increase indicates that repetitive inner speech has been used to support S–R transformations by assigning verbal labels to the presented visual stimuli^43^,^44^. Supporting this interpretation, a recent study showed that fronto-parietal regions disengage during short periods of verbal working memory maintenance, whereas an area located at the parietal–temporal boundary displayed stable activation, corresponding with the location of the nodes of the auditory network^45^. Thus, supporting the transformation of visual input into motor output, verbal representations of S–R associations might have been integrated into task processing via enhanced coupling of the DAN with the auditory network.

By automatizing responses during practice, the deactivation of the FPN allowed for an activation of the antagonistic DMN. Increasing activation of the DMN did not coincide with increasing error rates in the learning task, instead errors were slightly decreasing, in contrast to Eichele *et al*.^46^, see also Anticevic *et al*.^12^. This could be explained by an increasing segregation of the DMN from networks relevant for automatized task-processing, especially the CON. Possibly due to this segregation, activation in the DMN did not impair task performance, but instead putatively task-unrelated cognitive processes might have been instantiated in parallel to and independent of task-related processes. Consistent with the present results, a practice-related activation increase in the DMN after several days of practice was reported by Mason *et al*.^47^ Decreasing activation in subregions of the FPN, in combination with increasing activity in subregions of the DMN, was also reported by Chein and Schneider^31^ for longer practice sessions. Generally, the DMN is not only involved in off-task activities but can also support task-relevant, internally directed cognition^48^,^49^. However, since there is no obvious need for internally directed cognition during the instruction-based learning task, it seems to be more likely that task-irrelevant processes were instantiated in parallel to task-related processes than an active involvement of the DMN in task processing.

Several studies have explored the influence of the SAN^50^ on activation and connectivity of other brain areas. The majority of those studies reported a regulatory function for the SAN, and specifically for the anterior insula^51^,^52^,^53^. Especially, the SAN seems to be involved in suppressing DMN activation in tasks that require externally focused attention^54^,^55^. In contrast, Chen *et al*. found that parts of the FPN exhibited regulatory influence on DMN connectivity^56^. Liang *et al*. reported increased connectivity between the SAN and DMN, and the SAN and FPN, for task conditions with higher cognitive load^29^. In the present study, connectivity between the SAN and DMN decreased during practice, and concurrently DMN activation increased. In connection with the aforementioned literature, we are tempted to speculate that the regulatory influence of the SAN on the DMN was relaxed during practice, resulting in higher DMN activity. In addition, reduced coupling between the SAN and DMN during late practice could indicate some form of shielding of the DMN from task-relevant processes. Following this interpretation, and taking into account that the SAN is engaged slightly before other control networks^51^,^53^, the strengthened coupling between the SAN and CON might indicate that the SAN was putting the CON into an alert state at trial onset, to facilitate response execution when S–R transformation had been accomplished via the DAN–CON pathway. Adding to this line of reasoning, both the CON and SAN have nodes within anterior cingulate/medial superior prefrontal cortex and the anterior insula, with the SAN being located anterior to the CON^23^. The threefold connectivity increase within the CON and SAN, and between both networks could thus be seen as a tendency towards the unification of these anatomically similar networks during practice.

In the presented study, rapid learning was investigated by an instruction-based S–R learning task. While the ability to rapidly learn arbitrary associations between stimuli and responses constitutes an important component of human cognition, various other types of learning paradigms have been investigated that are also highly relevant for a general understanding of adaptivity in the brain. Specifically, a large body of literature is dedicated to procedural learning tasks involving more complex motor responses than used in the current study^57^. An interesting question is then to which degree the findings presented here generalize to those learning paradigms. As numerous studies have provided evidence for an engagement of the dorsal visual stream in the visual control of complex motor responses^58^,^59^, it is conceivable that the initial stage of learning in this domain might also be accompanied by enhanced coupling between the CON and DAN. In contrast, for longer timescales, it has been shown that procedural learning induces increased autonomy among task-related large-scale functional networks^4^. Given the dichotomy of short-term integration and long-term segregation of task-related networks during learning, characterizing this putative transition of large-scale connectivity will be crucial for a deeper understanding of adaptivity in the brain.

The findings presented here were based on a comparison between the learning task and a control task. This control task was carefully designed taking both theoretical and practical considerations into account. Using a working memory paradigm as a control task was theoretically motivated by prior studies, showing that working memory plays a crucial role during the early phase of instruction-based learning^13^,^26^. On the practical side, the control task was matched to the learning task in terms of stimulus material, timing of events and response options. However, to prevent any implicit S–R contingencies in the control task and to balance the number of left/right responses, the left/right responses were randomly assigned to 1-back equal/unequal judgements on each trial. This also led to generally increased RTs and different error rate changes in comparison to the learning task, which limits the comparability of the underlying cognitive and neural processes between the two tasks. From another point of view, however, behavioural performance changes were expected to be different between the learning task and the control task as specific learning-related changes (that is, decreasing RTs in combination with decreasing error rates) should only occur in the former. The finding that changes in brain activation and connectivity were more pronounced in the learning sample than in the control sample strongly suggests that significant group differences were primarily driven by features of the learning task. Yet, the specific design choices of a control task remain to some degree arbitrary, and these choices might influence the overall results. Further studies with varying (control) task designs might be useful to estimate to which degree the presented results generalize across specific task operationalizations. Follow-up studies could also investigate potential effects of relevant trait variables (for example, working memory capacity and various measures of intelligence) on rapid learning processes. While the large sample sizes employed in the present study render systematic group differences in relevant trait variables rather unlikely, no inferences could be made here about potential modulatory effects as these data were not collected.

A more technical limitation concerns the transformation of raw fMRI data into interpretable results. Specifically, whole-brain connectivity analyses depend on the definition of network nodes, which can be selected using some predefined atlas, or alternatively the nodes and networks can be determined based on the data at hand. Although the Power regions have been shown to be reliable regarding test–retest reliability and homogeneity^22^,^60^, the presented activation and connectivity results depend to a certain extent on the regions of interest (ROIs), the definition of networks and further steps of network construction. The advantage of predefined nodes and networks lies in good comparability to other studies that have used the same regions and networks. However, predefined networks do not allow for testing changes of the network structure, as it was performed for instance by Bassett *et al*.^4^ Yet, as we hypothesized that rapid learning processes might mainly evoke connectivity changes between cognitive control networks, we chose a set of regions that are representative for several established control networks.

Using large-scale networks for connectivity analyses can provide novel insights into the functional organization of the brain, based on the integrative view that remote brain regions collectively engage in task processing. In addition, this approach mitigates the multiple comparison problem in connectivity analysis, for instance by reducing the number of tests from 222 × 221/2=24,531 edges between 222 regions to 55 between/within-network-wise tests for 10 networks. However, as a drawback, local specificity is inherently reduced in this kind of analysis. Taking for example the increased mean connectivity between the relatively small CON (14 nodes) and the DAN (11 nodes), it would be a nontrivial task to determine a significant subset of the 154 edges that caused this increase without falling into statistical traps^61^. Moreover, investigating changes in large-scale functional connectivity can only shed light on the global organization of adaptive processes underlying learning in the brain, which originate from dynamic changes at finer spatial and temporal scales^62^.

Our findings contribute to the characterization of large-scale network dynamics in the human brain by investigating learning-induced connectivity changes. Previously, task-dependent changes of functional connectivity between these networks have been shown to be associated with long-term learning processes across hundreds of task repetitions and also for the non-repetitive reconfiguration of the current task space. In our work, we demonstrated that short-term task automatization during the first few practice trials of instructed novel tasks led to substantial and systematic changes in functional connectivity between these large-scale networks. This rapid functional network reorganization, based on global integration and segregation processes, together with complementary changes in activation dynamics, provided a comprehensive characterization of adaptive processes during short-term task automatization in the human brain. Generally, the findings support an integrative view of the brain, where task-dependent plasticity is not solely reflected by locally specific activation or connectivity changes but instead by systematic activation and connectivity changes between and within several large functional networks.

## Methods

### Samples

Two samples were used in this study, a learning sample (comprising *N*=70 subjects) and a control sample (1-back task, *N*=67). Sample sizes were chosen to ensure adequate power to detect medium-sized effects, based on a G*Power calculation (effect size *d*=0.5, type I error *α*=0.05, power 1−*β*=0.8), which resulted in a minimum sample size of 64 subjects for each group^63^. Data of the learning sample have been published before with a different focus (outcome-related)^64^,^65^. The learning sample originally consisted of two subgroups with different outcomes contingencies (*N*=2 × 35), which were collapsed in the current study, since the current study had its focus on practice-related effects irrespective of outcome contingencies. fMRI data of 71 subjects were collected and one subject was excluded from further analyses after quality control due to excessive head movement. As error rates were generally low for the learning task (all subjects<20% error rate), no subjects of the learning sample were excluded based on this behavioural measure. The control sample originally consisted of fMRI data of 73 subjects. Subjects were randomly sampled from the same population as the learning sample. Six of the 73 subjects showed high error rates (>20%) and were removed from further analyses to improve comparability of the control sample with the learning sample. The learning sample consisted of 44 females and 26 males with a mean age of 24.4 (s.d.=4.1) years. The control sample comprised 42 females and 25 males with a mean age of 23.9 (s.d.=3.3) years. Statistical tests confirmed that the two samples were balanced with respect to age (two-sample *t*-test, *P*=0.43, *t*=0.8, df=135) and gender (*χ*^2^-test, *P*=0.98). The experimental protocol was approved by the Ethics Committee of the Technische Unversität Dresden and conformed to the World Medical Association's Declaration of Helsinki. All participants gave written informed consent before taking part in the experiment and were paid €8 per hour for their participation or received course credit.

### Learning task

In the instruction-based learning task, subjects were asked to practice S–R associations between four symbols and two responses (left/right-hand index finger button presses). Before practice, S–R associations were explicitly instructed. To this end, an instruction screen was presented for 10 s showing four symbols simultaneously, two on the right side and two on the left side, with side indicating the required response. The instruction screen was followed by a sequence of single trials (practice phase). In each trial, one of the four symbols was presented and subjects had to respond as instructed. Symbols were presented in randomized order. Feedback was given after a response or after maximally 1.5 s. In case of a correct response, the symbol was highlighted in a colour for 0.5 s, whereas after an erroneous response (or after 1.5 s elapsed without response), the symbol was highlighted in grey. In case of an erroneous response (or miss), the trial was repeated, and both trials were excluded from analysis. Trials were jittered with randomized 0.8 or 3.5 s inter-trial intervals. From each symbol, eight correctly performed trials were collected, that is, 32 trials per stimulus set. The whole procedure (that is, instruction screen and practice phase) was repeated 20 times, each time using a novel set of symbols.

### 1-Back task

During task performance, learning effects can potentially occur in various domains and might also include more general adaptive processes that are not specific for practicing S–R rules. For instance, subjects may visually adapt to the specific symbols presented within each task block, or might get used to the general structure of the task (for example, trial timing, alternating instruction and practice phases and so on). To control for effects unspecific for practicing S–R rules, it was necessary to compare the learning task with a control task. This control task was instantiated in a separate, independent sample. The between-subject design was chosen to avoid any interference effects potentially associated with a within-subject design (for example, task-switching costs), and large sample sizes were provided accordingly.

The control task was designed such that basic features of the task (that is, stimulus material, motor responses and timing) were identical to the learning task. Hence, adaptive processes that occurred in these domains in the learning task were assumed to occur in the control task as well, and subtraction of the control task from the learning task was assumed to remove these effects of no interest. However, to serve as a control task for S–R learning, it was crucial to remove any S–R associations in the control task: if subjects were enabled to perform the control task by exploiting (hidden) S–R associations, they could potentially activate similar processes as in the learning task, and in this case, subtraction would remove the effects of interest.

As instructed S–R rules have to be held in working memory initially before being transformed into more pragmatic S–R associations during practice (cf. Meiran *et al*.^26^), we selected a control task that would, in contrast to the hypothesized release of working memory in the learning task, require a constantly high level of engagement of working memory-related processes.

These considerations motivated the choice of a 1-back task with randomized left/right response mappings as control task. In this 1-back task, subjects were also presented with 20 stimulus sets, each set consisting of four symbols. However, while temporal structure (that is, stimulus presentation/response window and jittering) and stimulus material were identical to the learning task, subject were instructed to perform a 1-back task during the single-trial phase. In each trial, subjects had to decide if the current symbol was identical to the preceding symbol or not. Erroneous responses were indicated but trials were not repeated to avoid response ambiguities. To remove any potential S–R associations and to balance the number of left/right responses, response mappings were cued randomly, that is, in each trial the equal/unequal symbols indicated left/right-hand responses. The 10 s starting screen did not instruct S–R associations but was still presented to visually familiarize subjects with the upcoming symbols. During the last second, one of the four symbols of the instruction screen was marked by a frame to indicate the preceding symbol to the first trial.

### Behavioural analysis

For the analysis of RT and error rate changes induced by practice, trials were assigned to repetition levels according to the number of a symbol's appearance. That is, all first appearances of the four symbols counted as repetition level 1 trials, all second appearances of the four symbols as repetition level 2 trails and so on until repetition level 8. We then defined early practice as the mean of repetition level 1 and 2 trials, and late practice as the mean of repetition level 7 and 8 trials. For RTs, erroneous trials were excluded and only correctly performed trials were taken into account. Furthermore, the very first trial of each practice phase was excluded, since these trials could be influenced by starting costs or switch costs occurring at the very beginning of practice. The analysis of the control task was identical to the analysis of the learning task.

While trial-and-error learning studies typically put their focus on error rates, we suggest RT decrease as the primary behavioural marker for instruction-based learning. First, we hypothesized that practice goes along with increased efficiency, and a putative RT speed-up of correctly performed trials would be an appropriate marker to confirm this hypothesis. Moreover, instruction-based learning was characterized by a very low error rate with 3.1% error (median) on average during early practice already, indicating that the instruction was presented successfully. The minor drop to 1.9% error (median) during late practice could be influenced by ceiling effects as well as superimposed reinforcement learning occurring due to feedback in case of erroneous responses, rendering error rates a suboptimal behavioural marker for efficiency increase.

Since the learning task had generally considerably faster RTs than the control task (581 ms compared to 839 ms overall mean values), we used relative RT decreases to compare the two samples. Relative RT decreases were computed as 100 × (1−RT_late_/RT_early_).

RT changes were normally distributed in both samples as assessed with the Shapiro–Wilk test (*P*=0.49 and *P*=0.18 for the learning and control sample, respectively). Error rate changes deviated from the normal distribution in the learning sample (*P*=0.002) but not in the control sample (*P*=0.063).

### fMRI scanning

Functional and structural images of both samples were acquired on the same Siemens 3 T Trio Scanner equipped with a 16-channel circularly polarized head coil. A gradient echo planar sequence with repetition time (TR)=2 s, echo time (TE)=30 ms and flip=80° was used for functional imaging. Volumes consisted of 26 slices with an in-plane resolution of 4 × 4 mm and a thickness of 5 mm. Presentation 12.0 (Neurobehavioral Systems) software was used to run the experiment. Structural images were also obtained but were only used for neuroradiological assessment in the current study.

### Preprocessing

Preprocessing of functional data from both samples was performed with SPM8 running in Matlab 7.12. Preprocessing consisted of slice-time correction, rigid body movement correction (three translation and three rotation parameters), normalization of the mean functional image to the SPM MNI EPI template (resampling to 3 × 3 × 3 mm resolution) and smoothing with a Gaussian kernel, full width at half maximum=8 mm.

### General linear model (GLM)

As in the behavioural analysis, practice trials were assigned to repetition levels 1 to 8. The linear model consisted of 12 design-related regressors plus the 6 movement regressors generated by SPM during standard movement correction. We used only these six movement regressors, since it has been shown that excessive use of nuisance regressors can remove network structure from fMRI data^66^. The 12 design regressors consisted of 8 event-related repetition level regressors plus 2 regressors for the instruction phase (one event-related and one with a duration of 10 s). Moreover, erroneous trials and first trials of practice phases were modelled with two separate regressors. The high-pass filter was set to a cutoff of 128 s, and the model was estimated with ordinary least squares (that is, AR(1) off). The model for the control task was the same as the model for the learning task.

### Regions of interest

ROIs were taken from Power *et al*.^23^ The Power regions have been shown to provide higher test–retest reliability for global and local network properties than the frequently used AAL atlas^60^. Of the originally 264 ROIs in Power *et al*., 227 were assigned to 10 brain networks in Cole *et al*.^2^ based on the originally 13 networks of Power *et al*. For each of these 227 ROIs, a sphere (or 3D cross) consisting of seven voxels (each voxel of size 3 × 3 × 3 mm^3^) was defined. For each subject, individual ROIs were included into analysis if at least five of the seven voxels were inside the SPM whole-brain mask. ROIs were completely excluded from analysis if <90% of the subjects of the learning sample had values in there. This procedure led to the exclusion of five ROIs, leaving 222 ROIs for the analysis, see also Supplementary Table 3.

Moreover, one large ROI (‘PowComp') was defined by taking the complement of the union of all 264 ROIs, where this time spheres consisted of 33 voxels. This procedure ensured that voxels in PowComp were sufficiently far away from the Power ROIs. The PowComp ROI was used to define a nuisance signal for connectivity analysis (see below).

### Activation analysis

For activation analysis, we defined contrast images for early and late practice as repetition levels 1+2 and repetition levels 7+8, respectively. For each of the 222 ROIs, mean values were extracted from the early and late contrast images. Then, mean values for the 10 networks were computed by averaging across all ROIs of each network. For statistical analysis, the change from early to late practice (that is, late–early) was computed for each network. Finally, two-sided two-sample *t-*tests (learning against control) were applied to the late–early network activation values. The *P* values were then Bonferroni-corrected for 10 tests. Moreover, activation changes of the learning sample were tested *post hoc* against zero (one-sample *t*-tests) to check if differences were indeed practice-related, and not only driven by differences between the learning and the control sample.

### Connectivity analysis

For connectivity analysis, mean residual time series from the GLM were extracted for each ROI. This procedure was motivated by Cao *et al*., who demonstrated that regressing out average task-related activity can improve test–retest reliability in fMRI connectivity analyses^60^.

Moreover, a mean time series from the PowComp ROI was extracted (see above), and regressed out of the 222 ROIs of interest. Please note that this (relaxed) ‘global signal regression' step does not induce circularity as described in Murphy *et al*.^67^ since the source voxels of the ‘global signal' are spatially separated from the ROIs of interest.

Besides the high-pass filter of the GLM no further filtering was applied, following the argument of Cole *et al*. that task-relevant frequency bands in fMRI have not yet been sufficiently characterized^2^. Furthermore, in a test–retest study, Braun *et al*. found that global signal regression and a broad frequency range provided highest reliability for various graph theoretical measures^68^.

Each practice phase comprised ∼47 functional volumes. The first two volumes were discarded to account for putative starting costs related to the first trial, in accordance with behavioural and activation analysis. Then, practice phases were cut into three equidistant sections with the first and last third representing early and late practice, respectively. Resulting time series windows of early and late practice had a length of ∼30 s, which can be sufficient for robust connectivity analysis^69^,^70^.

As a measure of functional connectivity, the Pearson correlation coefficient for each pair of ROIs and for each of the 20 practice phases was computed for early and late practice separately. The correlation coefficients were then Fisher *z*-transformed. No threshold was applied to the correlation coefficients, that is, resulting networks were weighted and signed. This was done in anticipation of repeated averaging of correlation coefficients across practice phases and sets of edges, which should be preferably done with normally distributed variables.

The 20 values per subjects for early and late practice were averaged across practice phases to obtain mean connectivity values for early and late practice, respectively. Mean connectivity between and within networks was computed as the average across all edges between/within those networks. The change of connectivity from early to late practice was computed for each pair of networks and within networks by computing the difference of late minus early practice. Finally, two-sided two-sample *t*-tests were applied to assess the statistical significance of connectivity change differences between the learning and the control task. The 55 tests were corrected for multiple testing using Bonferroni correction as well as the less conservative FDR correction. Moreover, connectivity changes of the learning sample were tested *post hoc* against zero (one-sample *t*-tests) to check if differences were indeed practice-related, and not driven by potential effects in the control sample.

Note that, given the above described analysis strategy, connectivity analyses were orthogonal to activation analyses, since activation analyses were based on the beta estimates of the GLM, whereas connectivity analyses were based on the residuals of the GLM.

Glass brain images and videos were generated with the BrainNet toolbox^71^. In images/videos with mainly decreasing connectivity, increasing edges were not shown (DMN–Visual: 486 of 1,674, 29.0%; DMN–CON: 40 of 756, 5.3%; DMN–SAN: 148 of 972, 15.2%; DMN–VAN: 107 of 486, 22.0%; DMN–Auditory: 67 of 702, 9.5%; FPN–DAN: 64 of 275, 23.3%), and in images/videos with mainly increasing connectivity, decreasing edges were not shown (CON–Visual: 81 of 434, 18.7%; within CON: 4 of 91, 4.4%; CON–SAN: 28 of 252, 11.1%; CON–DAN: 9 of 154, 5.8%; within SAN: 13 of 153, 8.5%; DAN–Auditory: 8 of 143, 5.6%).

### Subdivision

The CON and DAN were subdivided into smaller clusters of nodes based on their spatial arrangement. For the CON, four subclusters were defined, namely, anterior insula/frontal operculum, SMA/dorsal anterior cingulate cortex, midcingulate cortex and supramarginal gyrus, and for the DAN also four subcluster were defined, namely, precuneus/parietal cortex, temporal lobe, frontal eye fields and occipital cortex. For coordinates of the ROIs of each cluster see Supplementary Table 4. Pairwise mean connectivity values between subclusters were computed as the average across all connections for both samples and then the difference between the learning sample and the control sample was computed, leading to 16 values. The distribution of the values was tested for normality with the Shapiro–Wilk test.

### RT correlations with connectivity changes

The change of mean connectivity between the CON and DAN from early to late practice was correlated across subjects with relative RT speed-up from early to late practice. The Pearson correlation coefficient was computed for each sample and transformed into *z*-scores; the difference between the two samples was then tested for significance.

The same procedure was applied to connectivity changes of the 14 × 11=154 single edges between the CON and DAN. After Bonferroni correction for 154 tests, one connection was found significantly correlated with RT changes. As a *post hoc* test for this specific connection, the correlation coefficient of the learning sample was tested against zero to confirm a correlation between this connection and practice-related RT changes. The resulting *P* value was also corrected for 154 tests.

### Data availability

The data that support the findings of this study are available from the corresponding author on request.

## Additional information

**How to cite this article:** Mohr, H. *et al*. Integration and segregation of large-scale brain networks during short-term task automatization. *Nat. Commun.* **7,** 13217 doi: 10.1038/ncomms13217 (2016).

## Supplementary Material

Supplementary Information Supplementary Figures 1 - 5, Supplementary Tables 1 - 4 and Supplementary References

Supplementary Movie 1 3D-visualization of increasing functional brain connectivity between the cingulo-opercular network (CON) and the dorsal attention network (DAN).

Supplementary Movie 2 3D-visualization of increasing functional brain connectivity between the visual network and the cingulo-opercular network (CON).

Supplementary Movie 3 3D-visualization of increasing functional brain connectivity within the cingulo-opercular network (CON).

Supplementary Movie 4 3D-visualization of increasing functional brain connectivity between the dorsal attention network (DAN) and the auditory network.

Supplementary Movie 5 3D-visualization of increasing functional brain connectivity between the cingulo-opercular network (CON) and the salience network (SAN).

Supplementary Movie 6 3D-visualization of increasing functional brain connectivity within the salience network (SAN).

Supplementary Movie 7 3D-visualization of decreasing functional brain connectivity between the fronto-parietal network (FPN) and the dorsal attention network (DAN).

Supplementary Movie 8 3D-visualization of decreasing functional brain connectivity between the default-mode network (DMN) and the cingulo-opercular network (CON).

Supplementary Movie 9 3D-visualization of decreasing functional brain connectivity between the default-mode network (DMN) and visual network.

Supplementary Movie 10 3D-visualization of decreasing functional brain connectivity between the default-mode network (DMN) and the salience network (SAN).

Supplementary Movie 11 3D-visualization of decreasing functional brain connectivity between the default-mode network (DMN) and the auditory network.

Supplementary Movie 12 3D-visualization of decreasing functional brain connectivity between the default-mode network (DMN) and the ventral attention network (VAN).

## Figures and Tables

**Figure 1:**
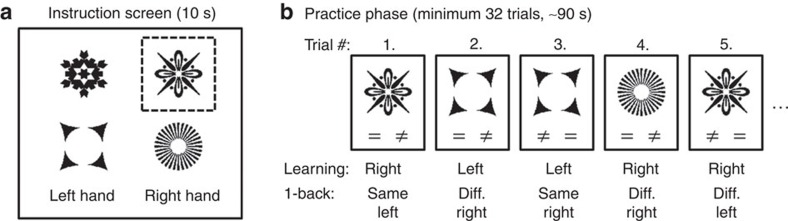
Design of the experimental tasks. In the learning task, subjects had to practice instructed stimulus–response (S–R) associations. (**a**) For instruction, subjects were presented with four symbols simultaneously for 10 s, indicating left/right-hand responses for each symbol. The dashed box was not presented in the learning task. (**b**) During the following practice phase, single symbols were presented sequentially and subjects had to respond to each symbol as instructed before. Equal/unequal signs were not presented in the learning task. This procedure was repeated 20 times featuring a novel stimulus set each time. As control task, a version of the 1-back task was implemented with stimulus material and temporal structure identical to the learning task. (**a**) Again, subjects saw four symbols on a screen, but without right/left-hand labelling. Instead, for the last second, a dashed box highlighted the reference symbol for the first trial of the following single-trial phase. (**b**) For the following sequence of trials subjects were asked to perform a 1-back task, that is, subjects had to decide if the current and preceding symbols were identical or not. Randomly chosen left/right to same/different mappings were indicated by respective signs presented below the stimulus symbols.
