# Supplementary material for: Integration and segregation of large-scale brain networks during short-term task automatization
Source: Nat Commun. 2016 Nov 3;7:13217. doi: 10.1038/ncomms13217 (PMC5097148; doi:10.1038/ncomms13217)
Supplement: Supplementary Information — Supplementary Figures 1 - 5, Supplementary Tables 1 - 4 and Supplementary References [file ncomms13217-s1.pdf]

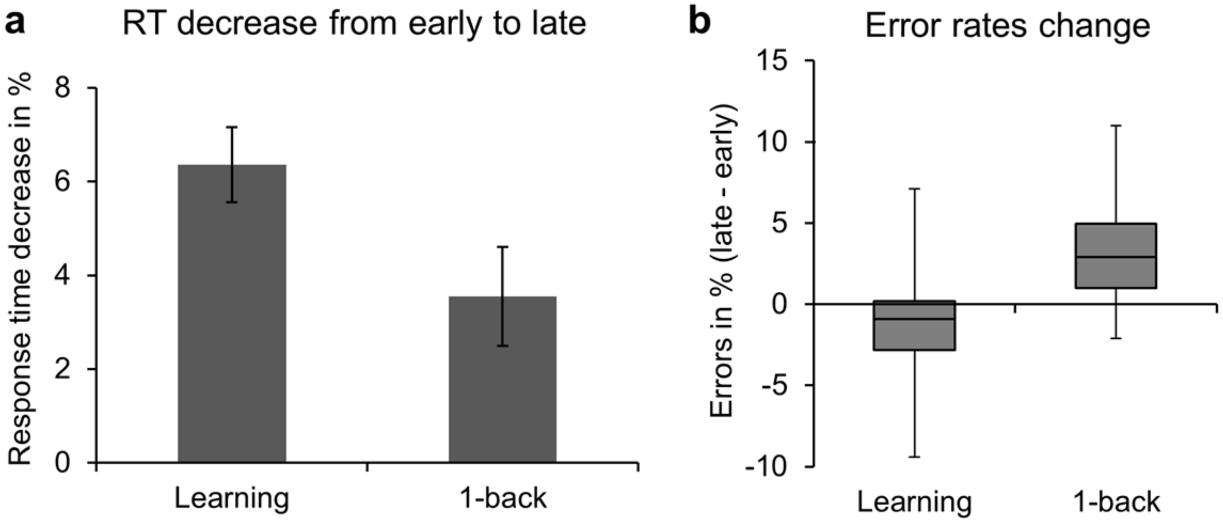

Supplementary Figure 1. Behavioral data for the learning task (N = 70) and the control task (N= 67). (**a**) Response time (RT) decreases from early to late practice. While subjects in the control task also accelerated responses during 1-back trials, the effect was larger for practice trials in the learning task. The y-axis represents the percentage of decrease of response times from early practice to late practice, computed as $100*(1 - RT_{late}/RT_{early})$. Using a two-sample t-test, the difference was found significant with $p = 4 \cdot 10^{-5}$. Black lines represent 95% confidence intervals. (**b**) Error rates difference between early and late (i.e. percentage $errors_{late}$ – percentage $errors_{early}$). Errors rates in the learning task were decreasing (median -0.9%, $p = 3 \cdot 10^{-5}$, Wilcoxon signed rank test), whereas in the control task errors rates increased across 1-back trials (median 2.9%, $p = 4 \cdot 10^{-10}$, Wilcoxon signed rank test; difference between the samples $p = 8 \cdot 10^{-15}$, Wilcoxon rank sum test). Vertical black lines indicate minimum and maximum values. Bars cover +-25% quantiles, and the horizontal black lines inside the bars indicate median values of the samples.

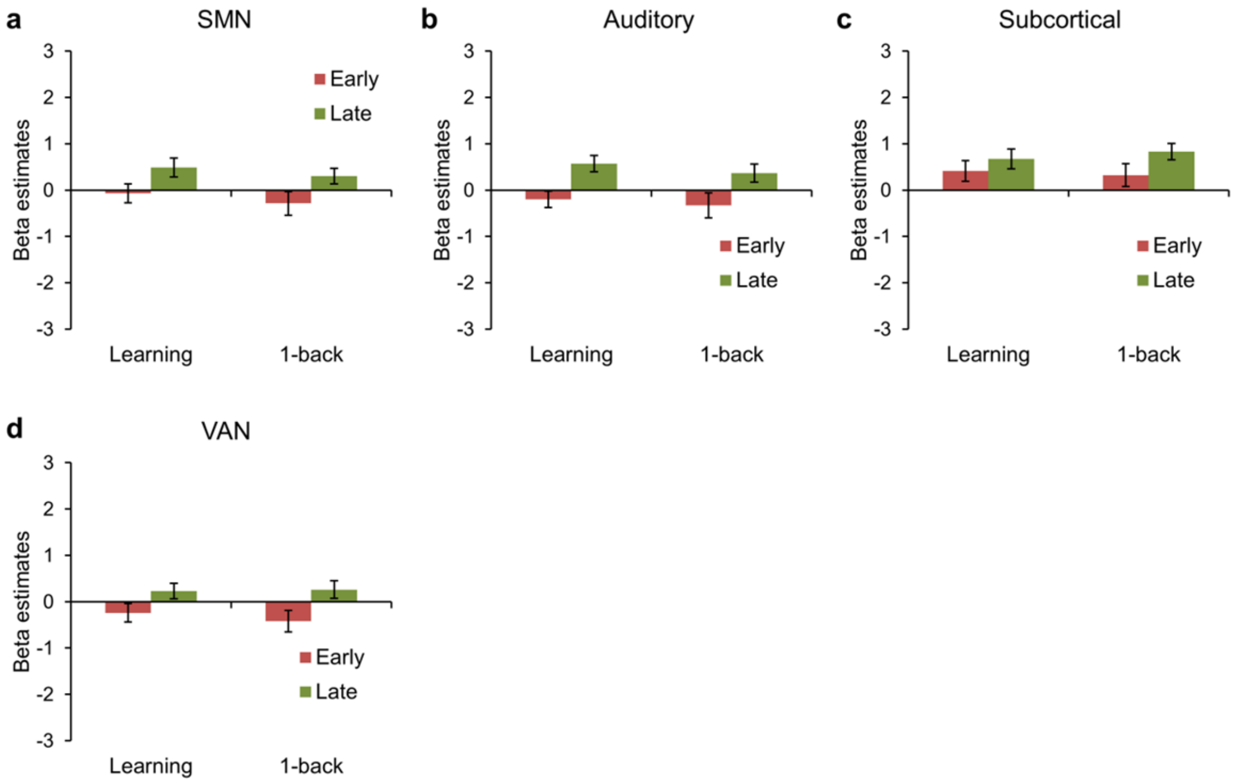

Supplementary Figure 2. Mean activations for 4 of the 10 networks for early and late practice and for the learning and control sample, respectively. (**a-d**) No significant differences of activation changes from early to late practice between the learning sample (N = 70) and the control sample (N = 67) were found in these 4 networks. Black lines represent 95% confidence intervals. P-values of two-sample and one-sample t-test can be found in Supplementary Table 1. Network abbr.: SMN: sensorimotor network, VAN: ventral attention network.

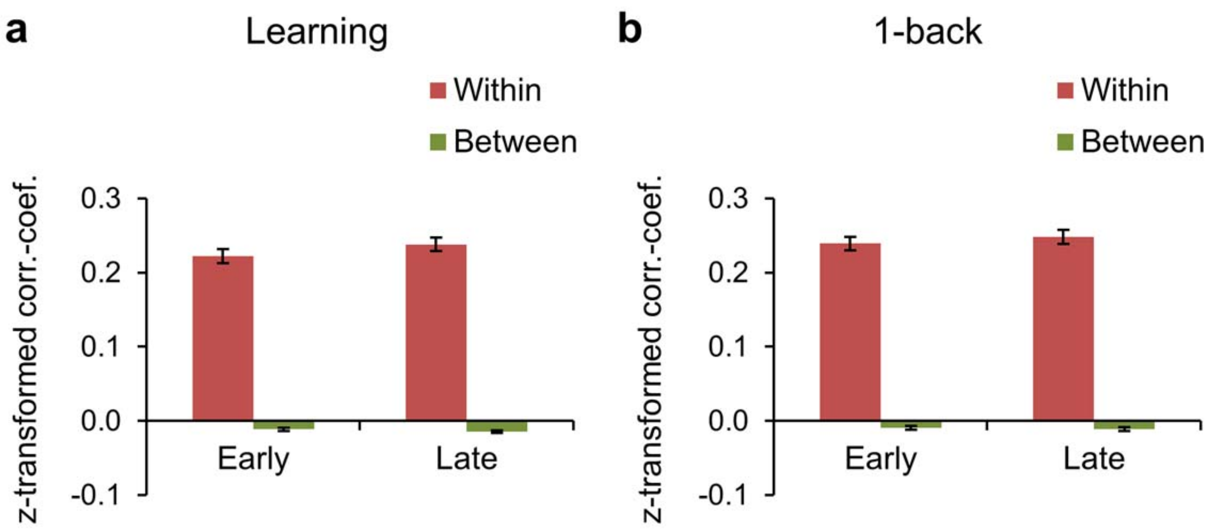

Supplementary Figure 3. Comparison of connectivity between edges that connect nodes within the same network and edges that connect nodes in different networks. The graph replicates the finding of Power et al.[1] that nodes within a network are more strongly connected than nodes of different networks. Black lines represent 95% confidence intervals. (**a**) Data of the learning sample (N = 70), (**b**) Data of the control sample (N = 67).

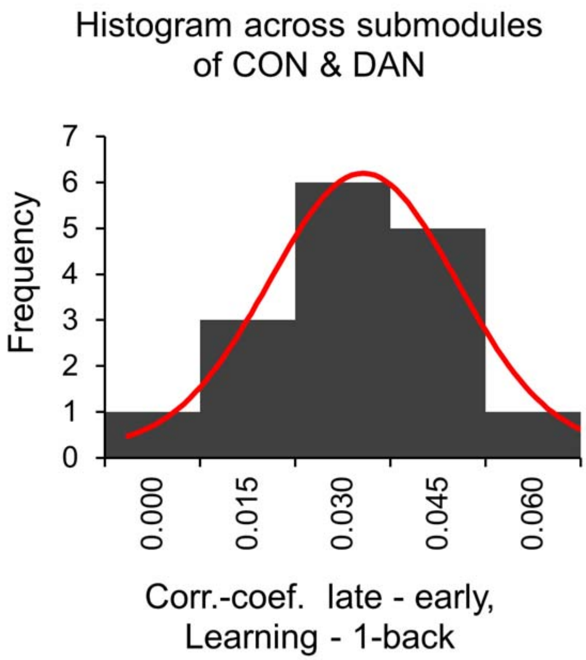

Supplementary Figure 4. Distribution of connectivity changes between subclusters of the CON and the DAN for the learning task (N = 70) minus the control task (N = 67). The increase from early practice to late practice followed a normal distribution across several spatially defined subclusters. Gray bars: histogram of the change of connectivity between early and late practice, learning minus control sample. Red line: fitted normal distribution.

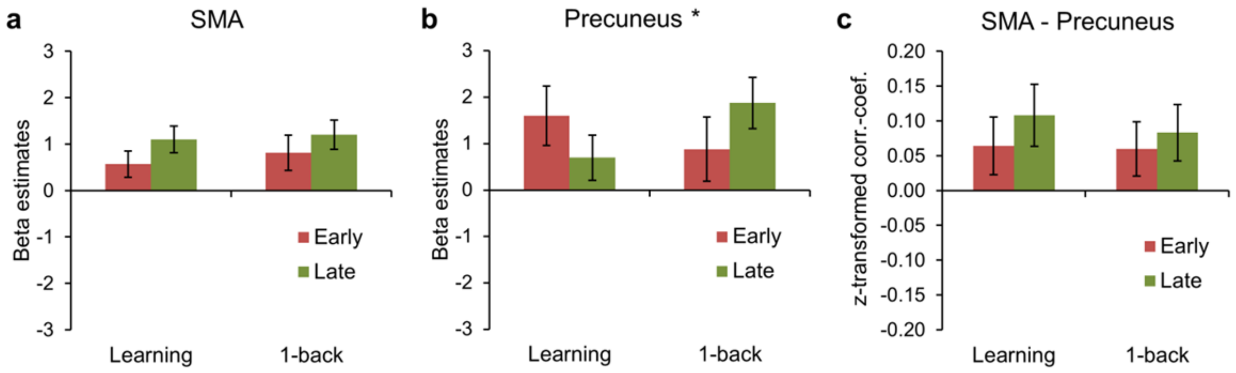

Supplementary Figure 5. Activation and connectivity for the two nodes where connectivity increase correlated with RT decrease in the learning task. (**a**) Activation profiles for the CON-based SMA node. This node showed increasing activation in the learning sample ($p = 3 \cdot 10^{-6}$, t = 5.1, df = 69) and in the control sample (p = 0.0003, t = 3.8, df = 66), with no difference between the samples (p = 0.36, t = 0.9, df = 135). (**b**) Activation for the precuneus node (part of the DAN). In this node, activation dropped for the learning task (p = 0.001, t = -3.4, df = 69) and increased for the control task ($p = 2 \cdot 10^{-5}$, t = 4.6, df = 66; difference between samples $p = 2 \cdot 10^{-7}$, t = -5.5, df = 135). (**c**) Connectivity between the two nodes. Connectivity change from early to late practice did not differ significantly between the learning and control sample (p = 0.26, t = 1.1, df = 135). Testing early against late practice revealed a significant connectivity increase in both samples (learning sample: p = 0.003, t = 3.0, df = 69; control sample: p = 0.042, t = 2.1, df = 66). Black lines represent 95% confidence intervals.

Supplementary Table 1. Activation differences from early to late practice for the learning sample and the control sample. All p-values are uncorrected; corrected p-values can be found in the results section of the main text. Cells marked in yellow indicate significant group differences after Bonferroni-correction. Abbreviations: DMN: Default Mode Network, FPN: Fronto-Parietal Network, SMN: Sensori-Motor Network, Subc.: Subcortical, CON: Cingulo-Opercular Network, SAN: Salience Network, VAN: Ventral Attention Network, DAN: Dorsal Attention Network.

	Learning vs. control, late - early		Learning sample, late - early		Control sample, late - early	
	P-values	T-values	P-values	T-values	P-values	T-values
DMN	0.0013	3.3	9.0E-21	13.3	5.1E-10	7.3
FPN	5.3E-10	-6.7	1.2E-15	-10.3	0.2653	-1.1
SMN	0.8260	-0.2	5.5E-10	7.2	1.2E-08	6.5
Visual	0.6182	0.5	2.6E-15	10.1	8.8E-13	8.8
Subc.	0.1157	-1.6	0.0184	2.4	1.4E-05	4.7
CON	0.3015	-1.0	1.8E-07	5.8	1.1E-09	7.1
SAN	0.0001	-4.1	0.2975	-1.0	2.9E-05	4.5
VAN	0.1084	-1.6	3.4E-06	5.1	2.9E-10	7.4
DAN	6.4E-06	-4.7	3.5E-05	-4.4	0.0293	2.2
Auditory	0.5175	0.6	1.2E-14	9.8	8.3E-12	8.3

Supplementary Table 2. Connectivity differences from early to late practice for the learning sample and the control sample. All p-values are uncorrected; corrected p-values can be found in the results section of the main text. Cells marked in yellow indicate significant group differences after Bonferroni-correction. Cells marked in orange indicate significant group differences after FDR-correction. Abbreviations: DMN: Default Mode Network, FPN: Fronto-Parietal Network, SMN: Sensori-Motor Network, Subc.: Subcortical, CON: Cingulo-Opercular Network, SAN: Salience Network, VAN: Ventral Attention Network, DAN: Dorsal Attention Network.

Learning sample vs. control sample, late – early, P-values

DMN	FPN	SMN	Visual	Subc.	CON	SAN	VAN	DAN	Auditory	
0.1157	0.0268	0.2666	0.0089	0.3536	3.6E-8	0.0002	0.0090	0.5007	0.0003	DMN
	0.1388	0.8011	0.0516	0.5845	0.0414	0.6524	0.9575	0.0005	0.0143	FPN
		0.9574	0.0295	0.7336	0.8792	0.9930	0.3143	0.0149	0.6279	SMN
			0.2527	0.4861	0.0034	0.0396	0.6671	0.9541	0.0532	Visual
				0.3805	0.8875	0.5560	0.5177	0.8543	0.4761	Subc.
					0.0041	0.0034	0.1771	4.2E-8	0.3747	CON
						0.0052	0.4984	0.3157	0.5227	SAN
							0.2280	0.2728	0.2372	VAN
								0.2834	0.0003	DAN
									0.4024	Auditory

Learning sample vs. control sample, late – early, T-values

DMN	FPN	SMN	Visual	Subc.	CON	SAN	VAN	DAN	Auditory	
1.6	2.2	-1.1	-2.7	0.9	-5.8	-3.9	-2.6	-0.7	-3.7	DMN
	-1.5	0.3	-2.0	-0.5	2.1	0.5	-0.1	-3.6	2.5	FPN
		-0.1	2.2	0.3	-0.2	0.0	1.0	2.5	-0.5	SMN
			1.1	0.7	3.0	2.1	0.4	0.1	2.0	Visual
				0.9	0.1	-0.6	0.6	0.2	-0.7	Subc.
					2.9	3.0	1.4	5.8	0.9	CON
						2.8	0.7	1.0	0.6	SAN
							1.2	1.1	1.2	VAN
								1.1	3.7	DAN
									0.8	Auditory

Learning sample, late – early, P-values

DMN	FPN	SMN	Visual	Subc.	CON	SAN	VAN	DAN	Auditory	
4.0E-5	0.0001	6.2E-6	3.9E-5	0.1860	4E-19	2E-11	8.8E-9	0.1972	4.4E-18	DMN
	0.0005	0.0210	9.3E-6	0.0750	3.7E-5	0.9438	0.0764	1.8E-8	1.7E-9	FPN
		0.0500	0.0001	0.2205	0.0900	0.3345	0.2869	6.0E-7	0.5263	SMN
			2.2E-6	0.2044	0.0012	0.0297	0.3538	0.0447	0.0005	Visual
				0.0024	0.4707	0.5956	0.4285	0.0276	0.7879	Subc.
					8E-10	4E-10	0.8316	1E-14	0.0019	CON
						3E-11	0.8396	0.0349	0.0078	SAN
							0.2340	0.0001	0.8053	VAN
								0.0653	2.7E-11	DAN
									0.0384	Auditory

Learning sample, late – early, T-values

DMN	FPN	SMN	Visual	Subc.	CON	SAN	VAN	DAN	Auditory	
4.4	4.1	-4.9	-4.4	1.3	-12.3	-8.0	-6.5	-1.3	-11.7	DMN
	-3.7	2.4	-4.8	-1.8	4.4	0.1	1.8	-6.4	6.9	FPN
		2.0	4.2	-1.2	1.7	1.0	1.1	5.5	0.6	SMN
			5.2	-1.3	3.4	2.2	0.9	2.0	3.7	Visual
				3.1	0.7	-0.5	0.8	-2.3	-0.3	Subc.
					7.1	7.3	0.2	9.8	3.2	CON
						7.9	-0.2	2.2	2.7	SAN
							1.2	4.1	-0.2	VAN
								1.9	7.9	DAN
									2.1	Auditory

Control sample, late – early, P-values

DMN	FPN	SMN	Visual	Subc.	CON	SAN	VAN	DAN	Auditory	
0.0041	0.1966	0.0020	0.1590	0.9553	0.0001	0.0024	0.0092	0.5940	1.2E-06	DMN
	0.0688	0.0725	0.0412	0.2532	0.1217	0.5487	0.0046	0.0022	0.0004	FPN
		0.0692	0.3011	0.1305	0.0643	0.3073	0.6942	0.1071	0.2008	SMN
			0.0023	0.0138	0.3790	0.4544	0.7887	0.0536	0.3222	Visual
				0.1146	0.6321	0.7630	0.8699	0.0245	0.4750	Subc.
					0.0512	0.0133	0.0938	0.3085	0.2320	CON
						0.0034	0.2377	0.3981	0.1034	SAN
							0.6252	0.0063	0.0689	VAN
								0.6042	0.0004	DAN
									0.5168	Auditory

Control sample, late – early, T-values

DMN	FPN	SMN	Visual	Subc.	CON	SAN	VAN	DAN	Auditory	
3.0	1.3	-3.2	-1.4	0.1	-4.0	-3.2	-2.7	-0.5	-5.3	DMN
	-1.8	1.8	-2.1	-1.2	1.6	-0.6	2.9	-3.2	3.7	FPN
		1.8	1.0	-1.5	1.9	1.0	-0.4	1.6	1.3	SMN
			3.2	-2.5	-0.9	-0.8	0.3	2.0	1.0	Visual
				1.6	0.5	0.3	-0.2	-2.3	0.7	Subc.
					2.0	2.5	-1.7	1.0	1.2	CON
						3.0	-1.2	0.9	1.7	SAN
							-0.5	2.8	-1.8	VAN
								0.5	3.7	DAN
									0.7	Auditory

Supplementary Table 3. Network nodes included in the network analyses. Coordinates and network assignments were taken from Power et al.[1] and Cole et al.[2]. Rows marked in gray were excluded from the analyses.

Network name	Network-No.
Default Mode Network	1
Fronto-Parietal Network	2
Sensori-Motor Network	3
Visual Cortex	4
Subcortical Areas	5
Cingulo-Opercular Network	6
Salience Network	7
Ventral Attention Network	8
Dorsal Attention Network	9
Auditory Network	10
Not assigned	NaN

Node-No.	MNI x	MNI y	MNI z	Network-No.	% Data Learning Sample	% Data 1-back Sample	Node-No. in Figure 3
1	-24	-99	-12	NaN			
2	27	-96	-12	NaN			
3	24	33	-18	NaN			
4	-57	-45	-24	NaN			
5	9	42	-24	NaN			
6	-21	-21	-21	NaN			
7	18	-27	-18	NaN			
8	-36	-30	-27	NaN			
9	66	-24	-18	NaN			
10	51	-33	-27	NaN			
11	54	-30	-18	NaN			
12	33	39	-12	NaN			
13	-6	-51	60	3	100.0	100.0	80
14	-15	-18	39	3	100.0	100.0	81
15	0	-15	48	3	100.0	100.0	82
16	9	-3	45	3	100.0	100.0	83
17	-6	-21	66	3	100.0	100.0	84
18	-6	-33	72	3	100.0	100.0	85
19	12	-33	75	3	100.0	100.0	86
20	-54	-24	42	3	100.0	100.0	87
21	30	-18	72	3	100.0	100.0	88
22	9	-45	72	3	100.0	100.0	89
23	-24	-30	72	3	100.0	100.0	90
24	-39	-18	54	3	100.0	100.0	91
25	30	-39	60	3	100.0	100.0	92

Node-No.	MNI x	MNI y	MNI z	Network-No.	% Data Learning Sample	% Data 1-back Sample	Node-No. in Figure 3
26	51	-21	42	3	100.0	100.0	93
27	-39	-27	69	3	97.1	100.0	94
28	21	-30	60	3	100.0	100.0	95
29	45	-9	57	3	100.0	100.0	96
30	-30	-42	60	3	100.0	100.0	97
31	9	-18	75	3	100.0	100.0	98
32	21	-42	69	3	100.0	100.0	99
33	-45	-33	48	3	100.0	100.0	100
34	-21	-30	60	3	100.0	100.0	101
35	-12	-18	75	3	100.0	100.0	102
36	42	-21	54	3	100.0	100.0	103
37	-39	-15	69	3	5.7	6.0	
38	-15	-45	72	3	100.0	100.0	104
39	3	-27	60	3	100.0	100.0	105
40	3	-18	57	3	100.0	100.0	106
41	39	-18	45	3	100.0	100.0	107
42	-48	-12	36	3	100.0	100.0	108
43	36	-9	15	3	100.0	100.0	109
44	51	-6	33	3	100.0	100.0	110
45	-54	-9	24	3	100.0	100.0	111
46	66	-9	24	3	100.0	100.0	112
47	-3	3	54	6	100.0	100.0	158
48	54	-27	33	6	100.0	100.0	159
49	18	-9	63	6	100.0	100.0	160
50	-15	-6	72	6	100.0	100.0	161
51	-9	-3	42	6	100.0	100.0	162
52	36	0	-3	6	100.0	100.0	163
53	12	0	69	6	100.0	100.0	164
54	6	9	51	6	100.0	100.0	165
55	-45	0	9	6	100.0	100.0	166
56	48	9	0	6	100.0	100.0	167
57	-33	3	3	6	100.0	100.0	168
58	-51	9	-3	6	100.0	100.0	169
59	-6	18	33	6	100.0	100.0	170
60	36	9	0	6	100.0	100.0	171
61	33	-27	12	10	100.0	100.0	210
62	66	-33	21	10	100.0	100.0	211
63	57	-15	6	10	100.0	100.0	212
64	-39	-33	18	10	100.0	100.0	213
65	-60	-24	15	10	100.0	100.0	214
66	-48	-27	6	10	100.0	100.0	215
67	42	-24	21	10	100.0	100.0	216
68	-51	-33	27	10	100.0	100.0	217
69	-54	-21	24	10	100.0	100.0	218
70	-54	-9	12	10	100.0	100.0	219
71	57	-6	12	10	100.0	100.0	220
72	60	-18	30	10	100.0	100.0	221
73	-30	-27	12	10	100.0	100.0	222
74	-42	-75	27	1	100.0	100.0	1
75	6	66	-3	1	10.0	9.0	

Node-No.	MNI x	MNI y	MNI z	Network-No.	% Data Learning Sample	% Data 1-back Sample	Node-No. in Figure 3
76	9	48	-15	1	95.7	94.0	2
77	-12	-39	0	1	100.0	100.0	3
78	-18	63	-9	1	1.4	4.5	
79	-45	-60	21	1	100.0	100.0	4
80	42	-72	27	1	100.0	100.0	5
81	-45	12	-33	1	98.6	97.0	6
82	45	15	-30	1	100.0	100.0	7
83	-69	-24	-15	1	2.9	4.5	
84	-57	-27	-15	NaN			
85	27	15	-18	NaN			
86	-45	-66	36	1	100.0	100.0	8
87	-39	-75	45	1	100.0	100.0	9
88	-6	-54	27	1	100.0	100.0	10
89	6	-60	36	1	100.0	100.0	11
90	-12	-57	15	1	100.0	100.0	12
91	-3	-48	12	1	100.0	100.0	13
92	9	-48	30	1	100.0	100.0	14
93	15	-63	27	1	100.0	100.0	15
94	-3	-36	45	1	100.0	100.0	16
95	12	-54	18	1	100.0	100.0	17
96	51	-60	36	1	100.0	100.0	18
97	24	33	48	1	100.0	100.0	19
98	-9	39	51	1	100.0	100.0	20
99	-15	30	54	1	100.0	100.0	21
100	-36	21	51	1	100.0	100.0	22
101	21	39	39	1	100.0	100.0	23
102	12	54	39	1	100.0	100.0	24
103	-9	54	39	1	100.0	100.0	25
104	-21	45	39	1	100.0	100.0	26
105	6	54	15	1	100.0	100.0	27
106	6	63	21	1	100.0	100.0	28
107	-6	51	0	1	100.0	100.0	29
108	9	54	3	1	100.0	100.0	30
109	-3	45	-9	1	100.0	100.0	31
110	9	42	-6	1	100.0	100.0	32
111	-12	45	9	1	100.0	100.0	33
112	-3	39	36	1	100.0	100.0	34
113	-3	42	15	1	100.0	100.0	35
114	-21	63	18	1	100.0	100.0	36
115	-9	48	24	1	100.0	100.0	37
116	66	-12	-18	1	92.9	85.1	38
117	-57	-12	-9	1	100.0	100.0	39
118	-57	-30	-3	1	100.0	100.0	40
119	66	-30	-9	1	100.0	100.0	41
120	-69	-42	-6	1	21.4	26.9	
121	12	30	60	1	100.0	100.0	42
122	12	36	21	1	100.0	100.0	43
123	51	-3	-15	1	98.6	100.0	44
124	-27	-39	-9	1	100.0	100.0	45
125	27	-36	-12	1	100.0	100.0	46

Node-No.	MNI x	MNI y	MNI z	Network-No.	% Data Learning Sample	% Data 1-back Sample	Node-No. in Figure 3
126	-33	-39	-15	1	100.0	100.0	47
127	27	-78	-33	1	91.4	100.0	48
128	51	6	-30	1	100.0	100.0	49
129	-54	3	-27	1	98.6	98.5	50
130	48	-51	30	1	100.0	100.0	51
131	-48	-42	0	1	100.0	100.0	52
132	-30	18	-18	NaN			
133	-3	-36	30	NaN			
134	-6	-72	42	NaN			
135	12	-66	42	NaN			
136	3	-48	51	NaN			
137	-45	30	-12	1	100.0	100.0	53
138	-9	12	66	8	100.0	100.0	190
139	48	36	-12	1	98.6	95.5	54
140	9	-90	-6	NaN			
141	18	-90	-15	NaN			
142	-12	-96	-12	NaN			
143	18	-48	-9	4	100.0	100.0	114
144	39	-72	15	4	100.0	100.0	115
145	9	-72	12	4	100.0	100.0	116
146	-9	-81	6	4	100.0	100.0	117
147	-27	-78	18	4	100.0	100.0	118
148	21	-66	3	4	100.0	100.0	119
149	-24	-90	18	4	100.0	100.0	120
150	27	-60	-9	4	100.0	100.0	121
151	-15	-72	-9	4	100.0	100.0	122
152	-18	-69	6	4	100.0	100.0	123
153	42	-78	-12	4	100.0	100.0	124
154	-48	-75	-9	4	98.6	100.0	125
155	-15	-90	30	4	100.0	100.0	126
156	15	-87	36	4	100.0	100.0	127
157	30	-78	24	4	100.0	100.0	128
158	21	-87	-3	4	100.0	100.0	129
159	15	-78	30	4	100.0	100.0	130
160	-15	-51	0	4	100.0	100.0	131
161	42	-66	-9	4	100.0	100.0	132
162	24	-87	24	4	100.0	100.0	133
163	6	-72	24	4	100.0	100.0	134
164	-42	-75	0	4	100.0	100.0	135
165	27	-78	-15	4	100.0	100.0	136
166	-15	-78	33	4	100.0	100.0	137
167	-3	-81	21	4	100.0	100.0	138
168	-39	-87	-6	4	100.0	100.0	139
169	36	-84	12	4	100.0	100.0	140
170	6	-81	6	4	100.0	100.0	141
171	-27	-90	3	4	100.0	100.0	142
172	-33	-78	-12	4	100.0	100.0	143
173	36	-81	0	4	100.0	100.0	144
174	-45	3	45	2	100.0	100.0	55
175	48	24	27	2	100.0	100.0	56

Node-No.	MNI x	MNI y	MNI z	Network-No.	% Data Learning Sample	% Data 1-back Sample	Node-No. in Figure 3
176	-48	12	24	2	100.0	100.0	57
177	-54	-48	42	2	100.0	100.0	58
178	-24	12	63	2	100.0	100.0	59
179	57	-54	-15	2	100.0	98.5	60
180	24	45	-15	2	98.6	97.0	61
181	33	54	-12	2	97.1	94.0	62
182	-21	42	-21	NaN			
183	-18	-75	-24	NaN			
184	18	-81	-33	NaN			
185	36	-66	-33	NaN			
186	48	9	33	2	100.0	100.0	63
187	-42	6	33	2	100.0	100.0	64
188	-42	39	21	2	100.0	100.0	65
189	39	42	15	2	100.0	100.0	66
190	48	-42	45	2	100.0	100.0	67
191	-27	-57	48	2	100.0	100.0	68
192	45	-54	48	2	100.0	100.0	69
193	33	15	57	2	100.0	100.0	70
194	36	-66	39	2	100.0	100.0	71
195	-42	-54	45	2	100.0	100.0	72
196	39	18	39	2	100.0	100.0	73
197	-33	54	3	2	100.0	100.0	74
198	-42	45	-3	2	100.0	100.0	75
199	33	-54	45	2	100.0	100.0	76
200	42	48	-3	2	100.0	100.0	77
201	-42	24	30	2	100.0	100.0	78
202	-3	27	45	2	100.0	100.0	79
203	12	-39	51	7	100.0	100.0	172
204	54	-45	36	7	100.0	100.0	173
205	42	0	48	7	100.0	100.0	174
206	30	33	27	7	100.0	100.0	175
207	48	21	9	7	100.0	100.0	176
208	-36	21	0	7	100.0	100.0	177
209	36	21	3	7	100.0	100.0	178
210	36	33	-3	7	100.0	100.0	179
211	33	15	-9	7	100.0	100.0	180
212	-12	27	24	7	100.0	100.0	181
213	0	15	45	7	100.0	100.0	182
214	-27	51	21	7	100.0	100.0	183
215	0	30	27	7	100.0	100.0	184
216	6	24	36	7	100.0	100.0	185
217	9	21	27	7	100.0	100.0	186
218	30	57	15	7	100.0	100.0	187
219	27	51	27	7	100.0	100.0	188
220	-39	51	18	7	100.0	100.0	189
221	3	-24	30	NaN			
222	6	-24	0	5	100.0	100.0	145
223	-3	-12	12	5	100.0	100.0	146
224	-9	-18	6	5	100.0	100.0	147
225	12	-18	9	5	100.0	100.0	148

Node-No.	MNI x	MNI y	MNI z	Network-No.	% Data Learning Sample	% Data 1-back Sample	Node-No. in Figure 3
226	-6	-27	-3	5	100.0	100.0	149
227	-21	6	-6	5	100.0	100.0	150
228	-15	3	9	5	100.0	100.0	151
229	30	-15	3	5	100.0	100.0	152
230	24	9	0	5	100.0	100.0	153
231	30	0	3	5	100.0	100.0	154
232	-30	-12	0	5	100.0	100.0	155
233	15	6	6	5	100.0	100.0	156
234	9	-3	6	5	100.0	100.0	157
235	54	-42	21	8	100.0	100.0	191
236	-57	-51	9	8	100.0	100.0	192
237	-54	-39	15	8	100.0	100.0	193
238	51	-33	9	8	100.0	100.0	194
239	51	-30	-3	8	100.0	100.0	195
240	57	-45	12	8	100.0	100.0	196
241	54	33	0	8	100.0	98.5	197
242	-48	24	0	8	100.0	100.0	198
243	-15	-66	-21	NaN			
244	-33	-54	-24	NaN			
245	21	-57	-24	NaN			
246	0	-63	-18	NaN			
247	33	-12	-33	NaN			
248	-30	-9	-36	NaN			
249	48	-3	-39	NaN			
250	-51	-6	-39	NaN			
251	9	-63	60	9	100.0	100.0	199
252	-51	-63	6	9	100.0	100.0	200
253	-48	-51	-21	NaN			
254	45	-48	-18	NaN			
255	48	-30	48	3	100.0	100.0	113
256	21	-66	48	9	100.0	100.0	201
257	45	-60	3	9	100.0	100.0	202
258	24	-57	60	9	100.0	100.0	203
259	-33	-45	48	9	100.0	100.0	204
260	-27	-72	36	9	100.0	100.0	205
261	-33	0	54	9	100.0	100.0	206
262	-42	-60	-9	9	100.0	100.0	207
263	-18	-60	63	9	100.0	100.0	208
264	30	-6	54	9	100.0	100.0	209

Supplementary Table 4. Subclusters of the CON and the DAN. Abbreviations: SMA/dACC: Supplementary Motor Area / dorsal Anterior Cingulate Cortex, SMG: Supramarginal Gyrus, MCC: Midcingulate Cortex, IC/fO: Insular Cortex / frontal Operculum, Prec./PC: Precuneus / Parietal Cortex, TL: Temporal Lobe, OCC: Occipital Cortex, FEF: Frontal Eye Fields

Cingulo-opercular network (CON):

MNI x	MNI y	MNI z	Region
-3	2	53	SMA/dACC
54	-28	34	SMG
19	-8	64	SMA/dACC
-16	-5	71	SMA/dACC
-10	-2	42	MCC
37	1	-4	IC/fO
13	-1	70	SMA/dACC
7	8	51	SMA/dACC
-45	0	9	IC/fO
49	8	-1	IC/fO
-34	3	4	IC/fO
-51	8	-2	IC/fO
-5	18	34	MCC
36	10	1	IC/fO

Dorsal attention network (DAN):

MNI x	MNI y	MNI z	Region
10	-62	61	Prec./PC
-52	-63	5	TL
22	-65	48	Prec./PC
46	-59	4	TL
25	-58	60	Prec./PC
-33	-46	47	Prec./PC
-27	-71	37	OCC
-32	-1	54	FEF
-42	-60	-9	TL
-17	-59	64	Prec./PC
29	-5	54	FEF

References

1. Power, J. D. *et al.* Functional Network Organization of the Human Brain. *Neuron* **72,** 665–678 (2011).
2. Cole, M. W. *et al.* Multi-task connectivity reveals flexible hubs for adaptive task control. *Nat. Neurosci.* **16,** 1348–1355 (2013).
